# Expanding the Swiss autosomal marker set to 32 STRs

**DOI:** 10.1007/s00414-021-02624-w

**Published:** 2021-06-18

**Authors:** Martin Zieger, Alexandre Gouy, Silvia Utz

**Affiliations:** 1 Institute of Forensic Medicine, Forensic Molecular Biology Department, University of Bern, Sulgenauweg 40, 3007 Bern, Switzerland; 2 Gouy Data Consulting, Sentier de Renges 4A, 1026 Denges, Switzerland

**Keywords:** Switzerland, STR, Investigator HDplex, Kinship, Population data

## Abstract

By genotyping 1198 individuals with the Qiagen Investigator® HDplex Kit, we expand the Swiss autosomal STR dataset to 32 loci, providing additional resources for complex kinship cases. We present the first high-quality allele frequency dataset for loci D2S1360, D5S2500, D7S1517, and D10S2325 that will be accessible through the ENFSI reference database STRidER. For loci D3S1744, D4S2366, D6S474, D8S1132, and D21S2055, we provide a first European STRidER dataset.

**Supplementary Information:**

The online version contains supplementary material available at 10.1007/s00414-021-02624-w.

The Qiagen Investigator® HDplex Kit provides valuable additional information to assess complex kinship scenarios, by supplementing the set of forensic standard short tandem repeat (STR) loci [1]. We genotyped 1198 Swiss individuals from a previous population study for those nine additional loci.

DNA extractions were prepared as described [2]. Multiplex PCR was performed in a reduced reaction volume of 12.5 μL. Capillary electrophoresis was run on a 3500xl genetic analyzer (ThermoFisher, USA) and data interpretation carried out with Genemapper® ID-X, v1.4 (Thermo Fisher, US). Details on the population structure can be found in [2]. Allele frequencies, forensic parameters, and test for Hardy–Weinberg equilibrium (HWE) with 10,000 permutations were calculated using STRAF [3], excluding two genotypes with three-allelic patterns. Results are listed in Table S1. None of the nine additional loci shows significant deviation from HWE. Allele frequencies are also available as Familias [4, 5] input file for all 32 loci (Table S2). Pairwise F_ST_ values for the six regional sub-populations defined in [2] revealed no intra-national differences, as expected (Table S3).

All genotype data for the three loci D12S391, D18S51, and SE33 are concordant with the data previously generated with Promega PowerPlex® Fusion 6C [2]. One sample showed a triplet in D6S474, another one in D10S2325. Five samples showed an almost complete allele dropout in marker D2S1360. This partial dropout has previously been observed and has been shown to be due to a SNP in the primer binding site [6]. Interestingly, in our dataset, all partial dropouts concerned allele 21, suggesting an association in the studied population of this allele with the SNP in the flanking region. We also detected a couple of off ladder alleles. All variants are listed in Table S4.

For marker D21S2055, heterozygote balance was usually below 60% if one allele consisted of less than 30 repeats and the other one of more than 30 repeats, limiting the utility of this marker for trace analysis and particularly DNA mixtures. However, there is no clear-cut allele length boundary for this phenomenon. We also observed pronounced peak imbalance between a couple of other pairs of alleles, e.g., 17.1/29 or 31/33. A similar observation has already been made by Tillmar et al. [7]. This suggests an association of a structural difference in the flanking regions with the STR repeat length, which might be worth a future investigation by sequencing.

Expanding the STR dataset increases the number of syntenic loci. Since ignoring linkage between loci might have an impact on certain kinship scenarios [8], we list the information for relevant syntenic loci, including loci distances based on Phillips et al. [9], in Table S5. We checked for linkage disequilibrium with the genepop R package [10, 11] by performing an exact test with 50,000 iterations and 1000 batches. For the complete dataset of 32 loci, we could not detect any significant linkage disequilibrium after Bonferroni correction (see Table S6).

The present dataset has passed the quality control for STRidER [12] and obtained the STRidER accession number STR000368.

## Supplementary Information

ESM 1 (XLSX 14.7 kb)

ESM 2 (TXT 7 kb)

ESM 3 (DOCX 12 kb)

ESM 4 (DOCX 13 kb)

ESM 5 (DOCX 21 kb)

ESM 6 (DOCX 948 kb)

## Declarations

Samples are the same as in [2]. All samples were collected with informed written consent. They were reversibly anonymized, to permit the donors to exert their right to withdraw their sample at any time. The Institute of Forensic Medicine, University of Bern, obtained the samples under an arbitrary number. The written consent documents with the names of the donors remained with the Red Cross. All documents distributed to the donors upon sampling were submitted to the responsible cantonal ethical committee and approval obtained.
