# Supplementary material for: Expanding the Swiss autosomal marker set to 32 STRs
Source: Int J Legal Med. 2021 Jun 18;135(6):2309–10. doi: 10.1007/s00414-021-02624-w (PMC8523451; doi:10.1007/s00414-021-02624-w)
Supplement: Supplementary file 3 — (DOCX 12 kb) [file 414_2021_2624_MOESM3_ESM.docx]

|  | **NW** | **CS** | **BE** | **TI** | **WS** |
| --- | --- | --- | --- | --- | --- |
| **SG** | 0.0015 | 0.0019 | 0.0013 | 0.0021 | 0.0015 |
| **WS** | 0.0011 | 0.0015 | 0.0011 | 0.0014 |  |
| **TI** | 0.0015 | 0.0021 | 0.0016 |  |  |
| **BE** | 0.0012 | 0.0012 |  |  |  |
| **CS** | 0.0013 |  |  |  |  |

Table S3: F_ST_ values between different regional Swiss subpopulations (WS = Western Switzerland; BE = Berne; NW = Northwestern Switzerland; CS = Central Switzerland; SG = St. Gallen; TI = Ticino).
