# Supplementary material for: Expanding the Swiss autosomal marker set to 32 STRs
Source: Int J Legal Med. 2021 Jun 18;135(6):2309–10. doi: 10.1007/s00414-021-02624-w (PMC8523451; doi:10.1007/s00414-021-02624-w)
Supplement: Supplementary file 4 — (DOCX 13 kb) [file 414_2021_2624_MOESM4_ESM.docx]

|  | **off-ladder alleles** | **dropout** | **triplet** |
| --- | --- | --- | --- |
| **D2S1360** | 18; 30.1 | 21 (5x, partial) |  |
| **D3S1744** | 10; 12 |  |  |
| **D4S2366** | 12.2 (2x) |  |  |
| **D5S2500** | 8 |  |  |
| **D6S474** |  |  | 13/14/16 |
| **SE33** | 15.3; *16.1; 27.3; 29.3* | *15; 16; 28.2* | 20/21/33 |
| **D7S1517** | 25.1 |  |  |
| **D8S1132** | 21.1 (2x); 23.2 |  |  |
| **D10S2325** | 5; 10.2; 12.2 (3x); 13.2; 20 |  | 7/(13)/14 |
| **D12S391** | 22.3; *28* | *22 (2x)* |  |
| **D18S51** | *22.1* |  |  |
| **D21S2055** | 28.3 |  |  |

Table S4: Variant alleles detected with Investigator® HDplex. All variants in italic letters were also detected with PowerPlex® Fusion 6C. The triplet in SE33 is also detected with PowerPlex® Fusion 6C and GlobalFiler™ and is not in the same sample than the triplet in D6S474. The three markers with grey background are part of PowerPlex® Fusion 6C and GlobalFiler™. The triplet at D10S2325 is largely unbalanced with allele 13 being significantly lower than the two other alleles.
