# Supplementary material for: Expanding the Swiss autosomal marker set to 32 STRs
Source: Int J Legal Med. 2021 Jun 18;135(6):2309–10. doi: 10.1007/s00414-021-02624-w (PMC8523451; doi:10.1007/s00414-021-02624-w)
Supplement: Supplementary file 5 — (DOCX 21 kb) [file 414_2021_2624_MOESM5_ESM.docx]

|  | PowerPlex® Fusion 6C | | | | | | | | | | | | | | | | | | | | | | | Investigator® HDplex | | | | | | | | |
| --- | --- | --- | --- | --- | --- | --- | --- | --- | --- | --- | --- | --- | --- | --- | --- | --- | --- | --- | --- | --- | --- | --- | --- | --- | --- | --- | --- | --- | --- | --- | --- | --- |
| **Marker** | D3S1358 | D1S1656 | D2S441 | D10S1248 | D13S317 | PentaE | D16S539 | D18S51 | D2S1338 | CSF1PO | PentaD | TH01 | vWA | D21S11 | D7S820 | D5S818 | TPOX | D8S1179 | D12S391 | D19S433 | SE33 | D22S1045 | FGA | D7S1517 | D3S1744 | D2S1360 | D6S474 | D4S2366 | D8S1132 | D5S2500 | D21S2055 | D10S2325 |
| **Position** | 3p21.31 | 1q42.2 | 2p14 | 10q26.3 | 13q31.1 | 15q26.2 | 16q24.1 | 18q21.33 | 2q35 | 5q32 | 21q22.3 | 11p15.5 | 12p13.31 | 21q21.1 | 7q21.11 | 5q23.2 | 2p25.3 | 8q24.13 | 12p13.2 | 19q12 | 6q15 | 22q12.3 | 4q31.3 | 7q31.33 | 3q24 | 2p24-p22 | 6q21-22 | 4p16-15.2 | 8q23.1 | 5q11.2 | 21q22 | 10p12 |
| **Same Chr.** |  |  |  |  |  |  |  |  |  |  |  |  |  |  |  |  |  |  |  |  |  |  |  |  |  |  |  |  |  |  |  |  |

**A**

Table S5: Syntenic loci. A) All syntenic loci among the 32 markers in the complete Swiss dataset of 32 autosomal STRs. Markers on the same chromosome are labelled by the same color. B) Syntenic loci on the same chromosomal arm with a distance smaller than 90cM. Genetic distances from Phillips et al. 2012 [9].

**B**

| **Pair of loci** | **Distance in cM** | **Recombination fraction (Kosambi)** | **Physical distance** |
| --- | --- | --- | --- |
| PentaD – D21S2055 | 9.9 | 0.098 | 3.9 Mb |
| vWA – D12S391 | 11.9 | 0.117 | 6.4 Mb |
| D8S1179 – D8S1132 | 16.5 | 0.159 | 18.6 Mb |
| SE33 – D6S474 | 23 | 0.217 | 23.9 Mb |
| D5S818 – CSF1PO | 27.8 | 0.252 | 26.3 Mb |
| D7S820 – D7S1517 | 31.9 | 0.282 | 39.7 Mb |
| TPOX – D2S1360 | 33.4 | 0.292 | 16 Mb |
| D21S11 – D21S2055 | 34.8 | 0.301 | 20.6 Mb |
| D21S11 – Penta D | 44.7 | 0.357 | 24.5 Mb |
| D2S1360 – D2S441 | 55.4 | 0.402 | 50.7 Mb |
| D5S2500 – D5S818 | 84.1 | 0.467 | 90.8MB |
| TPOX – D2S441 | 88.8 | 0.472 | 66.7 Mb |
