# Supplementary material for: Expanding the Swiss autosomal marker set to 32 STRs
Source: Int J Legal Med. 2021 Jun 18;135(6):2309–10. doi: 10.1007/s00414-021-02624-w (PMC8523451; doi:10.1007/s00414-021-02624-w)
Supplement: Supplementary file 6 — (DOCX 948 kb) [file 414_2021_2624_MOESM6_ESM.docx]

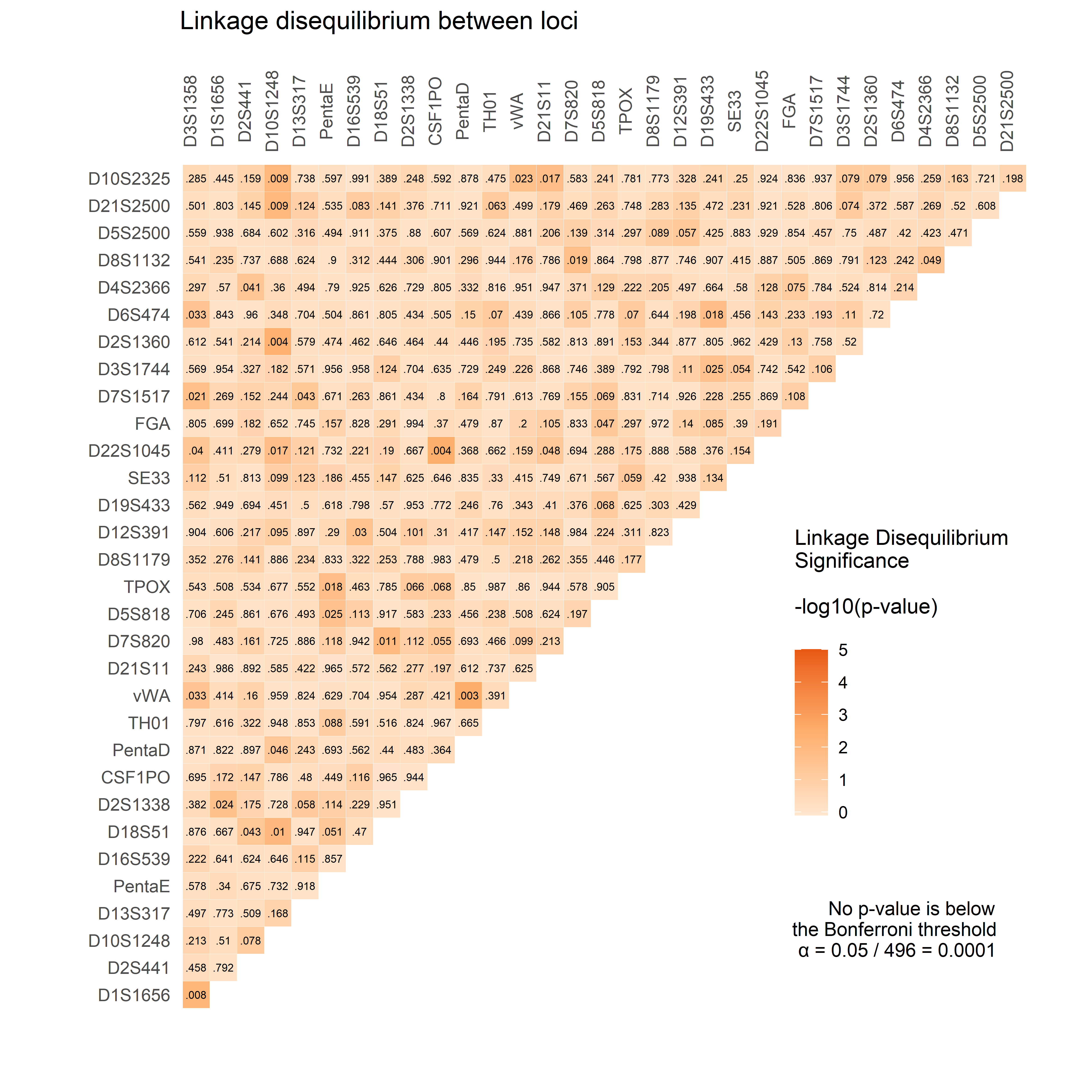


Table S6: p-value matrix for linkage disequilibrium, calculated for all 32 autosomal STR loci (Investigator® HDplex and PowerPlex® Fusion 6C) with 50'000 iterations on Genepop.
